# Analysis of the leaf transcriptome of *Musa acuminata* during interaction with *Mycosphaerella musicola*: gene assembly, annotation and marker development

**DOI:** 10.1186/1471-2164-14-78

**Published:** 2013-02-05

**Authors:** Marco A N Passos, Viviane Oliveira de Cruz, Flavia L Emediato, Cristiane Camargo de Teixeira, Vânia C Rennó Azevedo, Ana C M Brasileiro, Edson P Amorim, Claudia F Ferreira, Natalia F Martins, Roberto C Togawa, Georgios J Pappas, Orzenil Bonfim da Silva, Robert NG Miller

**Affiliations:** 1 Universidade de Brasília, Campus Universitário Darcy Ribeiro, Instituto de Ciências Biológicas, Departamento de Biologia Celular, CEP 70.910-900, Brasília, D.F, Brazil; 2 Universidade Católica de Brasília, SGAN 916, Módulo B, CEP 70.790-160, Brasília, D.F, Brazil; 3 EMBRAPA Recursos Genéticos e Biotecnologia, Parque Estação Biológica, CP 02372, CEP 70.770-900, Brasília, D.F, Brazil; 4 EMBRAPA Mandioca e Fruticultura Tropical, Rua Embrapa, CEP 44.380-000, Cruz das Almas, BA, Brazil

**Keywords:** Musa acuminata, Mycosphaerella musicola, Biotic stress, Transcriptome, 454 pyrosequencing, SSR

## Abstract

**Background:**

Although banana (*Musa* sp.) is an important edible crop, contributing towards poverty alleviation and food security, limited transcriptome datasets are available for use in accelerated molecular-based breeding in this genus. 454 GS-FLX Titanium technology was employed to determine the sequence of gene transcripts in genotypes of *Musa acuminata* ssp. *burmannicoides* Calcutta 4 and *M. acuminata* subgroup Cavendish cv. Grande Naine, contrasting in resistance to the fungal pathogen *Mycosphaerella musicola*, causal organism of Sigatoka leaf spot disease. To enrich for transcripts under biotic stress responses, full length-enriched cDNA libraries were prepared from whole plant leaf materials, both uninfected and artificially challenged with pathogen conidiospores.

**Results:**

The study generated 846,762 high quality sequence reads, with an average length of 334 bp and totalling 283 Mbp. *De novo* assembly generated 36,384 and 35,269 unigene sequences for *M. acuminata* Calcutta 4 and Cavendish Grande Naine, respectively. A total of 64.4% of the unigenes were annotated through Basic Local Alignment Search Tool (BLAST) similarity analyses against public databases.

Assembled sequences were functionally mapped to Gene Ontology (GO) terms, with unigene functions covering a diverse range of molecular functions, biological processes and cellular components. Genes from a number of defense-related pathways were observed in transcripts from each cDNA library. Over 99% of contig unigenes mapped to exon regions in the reference *M. acuminata* DH Pahang whole genome sequence. A total of 4068 genic-SSR loci were identified in Calcutta 4 and 4095 in Cavendish Grande Naine. A subset of 95 potential defense-related gene-derived simple sequence repeat (SSR) loci were validated for specific amplification and polymorphism across *M. acuminata* accessions. Fourteen loci were polymorphic, with alleles per polymorphic locus ranging from 3 to 8 and polymorphism information content ranging from 0.34 to 0.82.

**Conclusions:**

A large set of unigenes were characterized in this study for both *M. acuminata* Calcutta 4 and Cavendish Grande Naine, increasing the number of public domain *Musa* ESTs. This transcriptome is an invaluable resource for furthering our understanding of biological processes elicited during biotic stresses in *Musa*. Gene-based markers will facilitate molecular breeding strategies, forming the basis of genetic linkage mapping and analysis of quantitative trait loci.

## Background

Cultivated edible banana and plantains are derived from the progenitor species *Musa acuminata* Colla (A genome) and *Musa balbisiana* Colla (B genome), which are both members of the section *Eumusa*. These commodity fruit crops are amongst the most important across tropical and sub-tropical regions, contributing to food security, nutrition and poverty alleviation. Global annual production from over 120 countries on 5 continents is estimated to be approximately 102 million tonnes [1].

Global movement of *Musa* germplasm from its’centre of origin in Southeast Asia and the Pacific region has resulted in a spread of pests and disease, causing major constraints to banana production. Biotic stresses include fungi, bacteria, viruses, nematodes and insects. In excess of 40 fungal pathogens cause disease in banana [2], with three species of the genus *Mycosphaerella* recognized as important foliar pathogens. As causal members of the Sigatoka disease complex, *Mycosphaerella fijiensis* is responsible for black leaf streak disease, *Mycosphaerella musicola* for Sigatoka leaf spot disease, and *Mycosphaerella eumusae* for Eumusae leaf spot. *M. musicola* was the first *Mycosphaerella* pathogen to be recorded on banana, spreading from Java (Indonesia) in 1902 to most of the world’s production areas in the 1960s [3]. With a preference for higher altitudes and cooler temperatures, it is typically a greater problem during rainy seasons in subtropical banana growing regions [4,5]. Foliar necrotic lesions and diminished photosynthetic capacity cause reductions in fruit number and size per bunch, with premature fruit ripening observed in the field and post-harvest. Estimated production losses vary between 50 and 100%, given that infected fruits have no commercial value [6,7].

As disease resistance is absent in most cultivated varieties, in particular members of the Cavendish subgroup, control is largely based upon agronomic management practices and application of protectant and systemic fungicides. In addition to increasing production costs, long term dependence upon agrochemical control increases selection pressure for fungicide resistance or tolerance development in pathogen populations. Resistance to benzimidazole, triazole and strobilurin systemic fungicides has been reported in the genus *Mycosphaerella*[8,9].

Given the susceptibility to an ever-increasing range of pests and diseases, development of resistant cultivars through genetic improvement is of fundamental importance for sustainable disease management. In contrast to fertility in wild diploid *Musa* genotypes, commercial triploid and diploid cultivars are seedless and parthenocarpic, with fruit development via parthenocarpy. With maintenance of such plants by vegetative propagation [10], somatic mutation-driven evolution has resulted in a crop with a narrow genetic base, with many genotypes lacking resistance to pests and disease. Conventional genetic improvement is hindered principally by male and female sterility, with approaches time-consuming and demanding in terms of land use. For example, current hybridization strategies for the development of resistant tetraploid varieties rely upon sexually active wild or improved fertile *M. acuminata* diploids, which provide sources of resistance to biotic and abiotic stresses, for crossing with established semi-fertile triploid genotypes [11,12]. Success can be limited, however, given low numbers or absence of seeds. Complementary strategies for resolving constraints for *Musa* improvement are also under development, with molecular and tissue-culture approaches including mutagenesis, somaclonal variation, somatic hybridization and genetic modification via plant transformation (for review see [13]).

Genetic modification of *Musa* requires access to genomic information, including expression analysis of gene models under different conditions. Nuclear genome size has been reported to range from 534–615 Mbp in the genus, with variation observed between species and among *M. acuminata* genotypes [14]. The publication in July 2012 of a 90% complete draft of a reference whole genome sequence for a double haploid of *M. acuminata* ssp. *malaccensis* var. Pahang (DH Pahang) reported a genome size of 523 Mbp, with 36,542 predicted gene models [15]. Although banana is one of the world’s most important edible crops, comprehensive diverse transcriptome datasets, complementary to a whole genome reference sequence, are required for use in accelerated molecular-based breeding in this genus. Publically available datasets currently contain 15560 ESTs for *M. acuminata* and 5320 for *M. balbisiana* (accessed July 2012), numbers which represent only a fraction of the total number of unigene sequences expected to be present in the whole transcriptome. Examples of developed datasets include those from different genotypes, plant tissues [16] and during ripening [17,18]. Examination of gene expression in relation to drought tolerance has also been reported [19-21]. Only limited analysis of gene expression in response to fungal biotic stresses has been reported. Examples for economically important pathosystems include Musa-*Fusarium oxysporum* f. sp. *cubense*[22] and Musa-*M. fijiensis*[15,23,24].

Next Generation Sequencing (NGS) of uncloned cDNA is appropriate for whole genome transcriptome characterization and gene discovery. Today’s available 454 GS FLX platform with Titanium chemistry allows for read lengths of 400 bases, such that accurate *de novo* assembly of transcripts can be achieved. 454 transcriptome pyrosequencing has now been conducted in numerous important plants including *Arabidopsis thaliana*[25], *Oryza longistaminata*[26]*Medicago sativa*[27] and *Phaseolus vulgaris*[28]. 454 transcriptome analysis in plant-fungi pathosystems is also now being reported, for example in [29-33].

In order to develop a functional genomics resource for *M. acuminata*, including transcriptome response data in relation to infection by the fungal pathogen *M. musicola*, we performed Roche 454 Pyrosequencing of expressed genes in genotypes contrasting in resistance to Sigatoka leaf spot disease. Total RNA was extracted from whole plant leaf material from the wild diploid genotype *M. acuminata* ssp. *burmannicoides* Calcutta 4 (incompatible response) and the commercial triploid *M. acuminata* subgroup Cavendish cv. Grande Naine (compatible response), both uninfected and challenged with the pathogen. Transcriptome datasets were also exploited for large scale gene-based marker development.

## Results and discussion

The objectives of this work were to generate a transcriptome resource for *M. acuminata* which includes genes expressed in banana-*M. musicola* interactions using highly susceptible (Cavendish Grande Naine) and completely resistant (Calcutta 4) genotypes. Calcutta 4 is a wild fertile diploid widely employed in breeding programs for improvement of commercial cultivars (e.g. [34]). As a donor species, it is considered an important source of resistance to important fungal pathogens and nematodes. Given this importance, it has been adopted as a model for comparative genomics with rice [35,36], with functional genomics applications [19] and candidate resistance gene discovery also reported [37,38]. Cavendish subgroup bananas, such as Grande Naine, by contrast, are sterile triploids, which, although representing more than 40% of global production, lack resistance to biotic stresses, such that regular pesticide application is necessary for commercial production. In addition to unigene discovery for each genotype during this pathosystem interaction, large scale isolation of microsatellites and genic-SSR marker development was also conducted, for application in genetic mapping, genotyping and marker-assisted selection of specific traits in breeding populations.

### 454 sequencing statistics and assembly

Emulsion PCR and 454 pyrosequencing were conducted according to Roche standard protocols using GS FLX technology and Titanium series chemistry. Each cDNA library was sequenced on a ¼ segment of a single plate run, generating 978,133 raw sequence reads for the two genotypes, totalling over 466 megabases of sequence data. Following adaptor sequence trimming and short read (< 50 bp) removal, a total of 846,762 high quality reads (283 megabases) were processed. Table 1 shows a summary of size distribution for both genotype datasets.

**Table 1:** **Size distribution of the high quality *M. acuminata *Calcutta 4 and Cavendish Grande Naine 454-derived sequence reads**

	**M. acuminata Calcutta 4**			
EST Length summary (bp)	Min	Mean	Median	Max
	40	332	353	908
EST Length distribution(bp)	40 – 100	20472		
	101 – 200	39009		
	201 – 300	78901		
	301 – 400	152325		
	401 – 500	129795		
	>500	3737		
	**M. acuminata Cavendish Grande Naine**			
EST Length summary (bp)	Min	Mean	Median	Max
	40	336	362	1161
EST Length distribution(bp)	40 – 100	22665		
	101 – 200	38635		
	201 – 300	71477		
	301 – 400	140099		
	401 – 500	143815		
	>500	5832		

**Figure 1:**
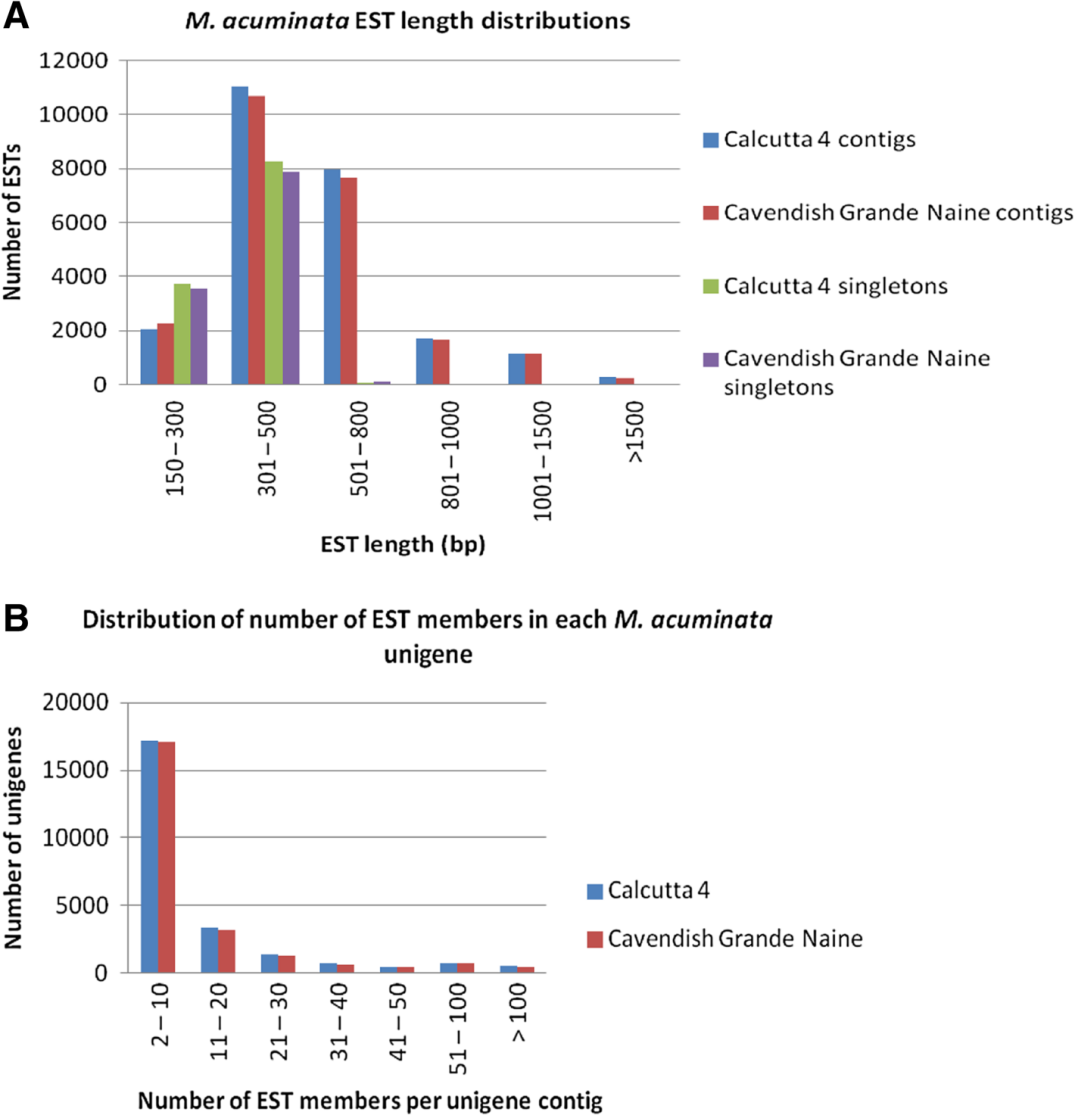
**Summary of sequence assemblies from *M. acuminata *Calcutta 4 and Cavendish Grande Naine datasets. A**, Length distribution of the assembled *M. acuminata* unigene contigs and singletons; **B**, Distribution of number of sequence reads in assembled *M. acuminata* unigene contigs.

Calcutta 4 sequence reads were *de novo* assembled into 36,384 unique unigene sequences, which included 24,259 contigs and 12,125 singletons. Of these, a total of 25,381 unigenes were represented in the transcriptome dataset from non-infected leaves and 25,154 in the dataset from pathogen-challenged leaves. In the case of Cavendish Grande Naine sequences, a similar assembly pattern was observed, with a total of 35,269 unigenes, composed of 23,729 contigs and 11,540 singletons. 18,611 unigenes were represented in the transcriptome dataset from non-infected leaves and 29,223 in the dataset from pathogen-challenged leaves. Singleton sequences which represent unique low level transcripts were generally of sufficient length to enable annotation, with an average length of 343 bp in Calcutta 4 and 345 bp in Cavendish Grande Naine. Unigene contig length distributions for each genotype are shown in Figure 1A. Average contig lengths of 552 bp and 548 bp were observed for Calcutta 4 and Cavendish Grande Naine, respectively. Distribution of the number of reads in a unigene contig (depth of a contig) were also similar in both assembled sequence datasets (Figure 1B).

Considering both contigs and singletons, the total number of genes per genotype corresponds well with the most recent estimate of 36,542 protein-coding gene models in the reference genome sequence for *M. acuminata* ssp. malaccensis var. Pahang (DH Pahang) [15], which may reflect the stringent quality analysis and assembly parameters adopted. It must be recognized, however, that *de novo* assembly may over-estimate gene numbers as a result of non-overlapping sequence read members present for a single gene. Alignment of contigs against the reference genome revealed such examples, with 24,097 of the 24,259 contigs in Calcutta 4 (99.3%) mapping to 16,519 gene models, and similarly 23,548 of the 23,729 contigs in Cavendish Grande Naine (99.2%) mapping to 16,402 reference genome gene models (Additional files 1 and 2).

The high percentages of unigenes mapped to gene model exons validates both the *de novo* contig assemblies and gene annotation of the reference *M. acuminata* genome, overlaying important information in relation to host expression during this plant-pathogen interaction. Through TBLASTX analysis of unmapped contigs, positive hits to genes in the NCBI EST (others) database identified a further 162 potential unigenes in Calcutta 4 and 181 in Cavendish Grande Naine (Additional file 1). Although these unaligned gene sequence may be specific to the *M. acuminata* genotypes, this data may also indicate additional genes requiring further curation in the reference genome.

In order to gain insights into broad similarities and differences between transcriptome unigene datasets (contigs and singletons) for the two *M. acuminata* genotypes evaluated in this study, all gene models in the reference genome were used as a base for identification of common and distinct genes. A Venn diagram (Additional file 3) illustrates overlap between the genotypes, with 16,386 common gene models identified to which mapped coverage of the query unigene sequence was greater than 90% and the percentage identity of the sequence relative to the genome was greater than 95%. This number represents 82.0% of the mapped gene models for each genotype. Although mapped unigenes specific to each genotype may be attributed to different evolutionary distances from *M. acuminata* ssp. malaccensis var. Pahang, overlap between data sets is likely to correlate with 454 sequence coverage.

All sequence data from the study are available for each genotype in the Sequence Read Archive (SRA) at the National Center for Biotechnology Information (NCBI) (submission SRA055816).

### Functional annotation and classification

Annotation of assembled unigene sequences was conducted by sequence similarity searches against the NCBI non-redundant protein sequence database (nr). BLASTX criteria were that the alignment length should be greater than 100 amino acids and the E-value cut-off at 10^-5^. Of the total estimated unigene sequences for Calcutta 4, 10,080 displayed significant identity to genes encoding proteins with known or putative function, 1,633 to genes encoding proteins with unknown function, and 13,513 showed no significant identity to any sequences in the database. Similar results were observed for Cavendish Grande Naine, with 10,645 unigenes displaying significant identity to genes encoding proteins with known or putative function, 1,800 to genes for proteins with unknown function, and 11,971 showing no significant identity to any database sequences. Unigene sequences and Blast annotations are summarized in Additional file 4. The protein domain-searching tool InterProScan (http://www.ebi.ac.uk/Tools/InterProScan/) was used to further annotate sequences. A total of 14,826 Calcutta 4 unigenes contained interpro domains, with 3,949 distinct domains represented in the unigene set. Of the 13,513 translated sequences with no significant Blast hits, 192 showed functional protein domains. Similarly, 14,006 Cavendish Grande Naine unigenes possessed interpro domains, with a total of 3,974 interpro domains represented. A total of 223 out of the 11,971 sequences with no significant hits to Genbank database sequences were identified with interpro domains. The most representative domains in both unigene datasets are summarized in Additional file 5. Novelty rates for unigene datasets based upon Blast and interpro analyses were 36.6% in the case of Calcutta 4 and 33.3% for Cavendish Grande Naine.

**Figure 2:**
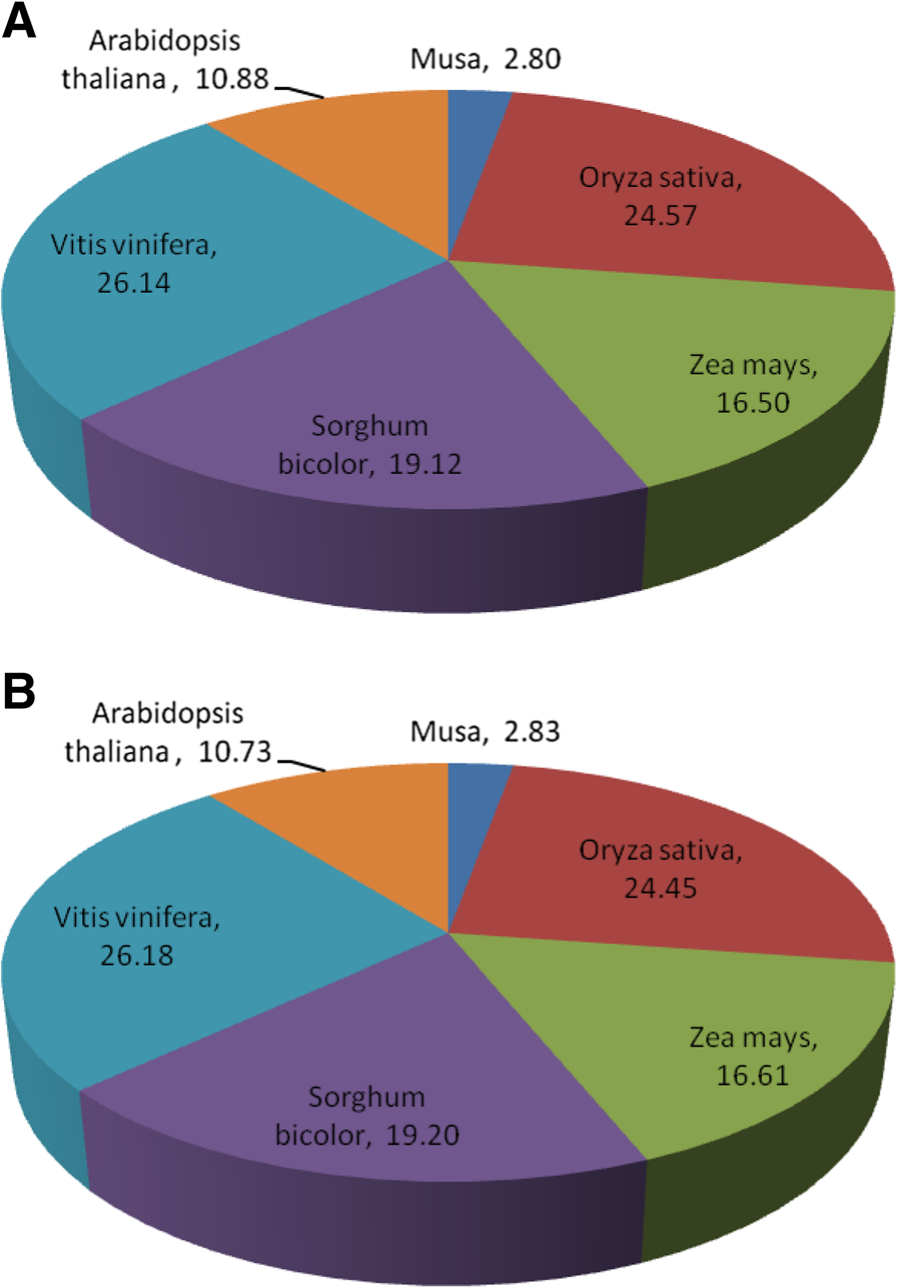
**Species distribution of *M. acuminata *unigenes shown as the percentage of the total homologous plant sequences. A**: *M. acuminata* Calcutta 4 unigenes; **B**: *M. acuminata* Cavendish Grande Naine unigenes. The best Blast hits of each sequence were analyzed.

Unigene sequences with significant homology to known plant proteins from the public database were abundant. On the basis of best blast hit analyses, respectively for Calcutta 4 and Cavendish Grande Naine unigenes, totals of 8,518 and 8,948 matched genes in rice (*Oryza sativa)*, 5,718 and 6,078 matched genes in maize (*Zea mays)*, 6,626 and 7,026 matched genes in sorghum (*Sorghum bicolor)*, 9,060 and 9,579 matched genes in grape (*Vitis vinifera)*, and 3,771 and 3,927 matched genes in *Arabidopsis thaliana* (Figure 2). Similar distributions were observed for *M. acuminata* DH-Pahang gene families [15]. For both Calcutta 4 and Cavendish Grande Naine datasets, only 2.8% of Blast hits to homologous plant sequences matched those from the GenBank NR protein database with a taxonomic filter for the genus *Musa*, indicating that our data represents a considerable contribution to expressed unigenes for the genus.

**Figure 3:**
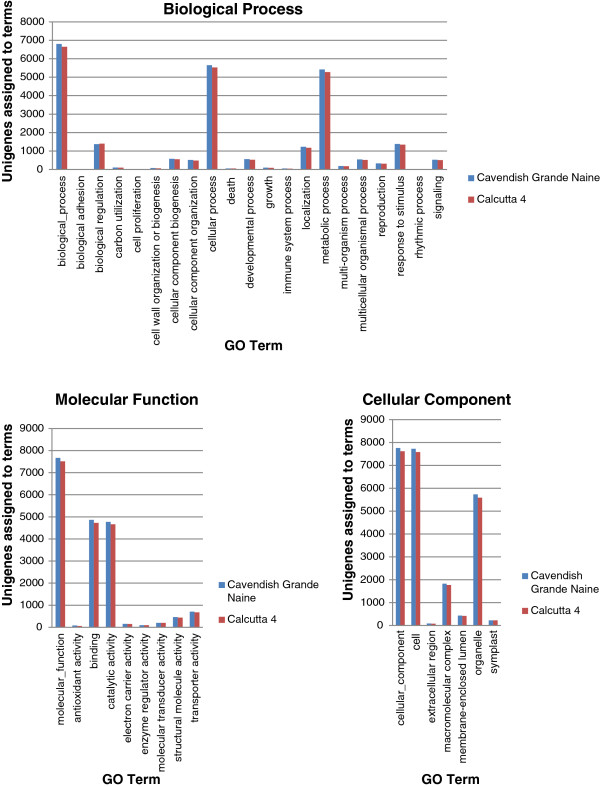
**Histogram presentation of Gene Ontology (GO) annotation of *M. acuminata *Calcutta 4 and Cavendish Grande Naine unigenes.** GO level 1 and 2 data are summarized in three main categories: biological process, molecular function and cellular component.

Functional classification of unigene sets was conducted through Gene Ontology (GO) assignment using Blast2GO. Data was categorized for each genotype across the three main GO categories of biological process, cellular component and molecular function. Calcutta 4 unigenes were assigned a total of 341,244 GO term annotations, with 162,468 biological process terms representing 10 GO levels, 69,390 molecular function terms from 10 levels and 109,386 cellular component terms from 9 levels. Similar assignments were seen with Cavendish Grande Naine unigene data, with 351,220 GO terms assigned across the three main categories. A total of 168,015 biological process terms were assigned, 70,878 molecular function terms and 112,327 cellular component terms. Within the biological process category, At GO level two, the majority of unigenes were assigned to “cellular process” (5,530 for C4, 5,649 for CAV), metabolic process (5,276 for C4, 5,418 for CAV), “biological regulation” (1,397 for C4, 1,377 for CAV), “response to stimulus” (1,343 for C4, 1,383 for CAV), and “localization” (1,179 for C4, 1,227 for CAV). Similarly, for Molecular Function, terms “binding” (4,732 for C4, 4,863 for CAV), “catalytic activity” (4,659 for C4, 4,771 for CAV) and “transporter activity” (678 for C4, 708 for CAV) were the most abundant assigned terms for the unigene datasets from both genotypes. Across the cellular function category, the most abundant terms were “cellular component” (7,620 for C4, 7,759 for CAV), “cell” (7,580 for C4, 7,725 for CAV), “organelle” (5,589 for C4, 5,735 for CAV) and “macromolecular complex” (1,772 for C4, 1,829 for CAV). Figure 3 summarises level 1 and 2 GO annotation of unigenes.

### *Musa*-*Mycosphaerella* interactions

*M. musicola* is a hemibiotrophic pathogen which penetrates leaf tissues through stomatal pores, following a period of epiphytic growth on the leaf surface. Once inside the host, the pathogen colonizes the intercellular space within mesophyll tissue layers and palisade tissues, without forming haustoria or infecting host cells. This biotrophic phase can last for a number of weeks before the onset of symptoms of necrotic lesions in mesophyll cells. Growth of conidiophores out through stomata then enables conidiospore development on the leaf surface. Notable differences in disease development are seen in totally resistant genotypes, with early necrosis of stomatal guard cells and death of only a limited number of host cells at the site of infection. No fungal sporulation is observed in such incompatible interactions.

Analysis of gene expression during banana-*Mycosphaerella* interactions has been limited to date [15,23,24], with our study, to the authors knowledge, the first massal transcriptome analysis for the *M. acuminata-M. musicola* pathosystem. A sequencing strategy for identification of transcripts from pathogen-challenged and unchallenged leaves was employed, with the preparation of two cDNA libraries per genotype (infected and non-infected). Given reports that during the early biotrophic phase of infection of banana with *M. fijiensis*, germ tubes penetrate stomata from 3 to 6DAI [39], a similar timecourse was employed for the *M. acuminata-M. musicola* pathosystem.

The use of detached leaf tissues in disease bioassays with *Mycosphaerella* banana pathogens has been reported on occasion to give inconsistent results (e.g. [40,41]), with development of disease symptoms not always correlating with those typically observed on intact plants. Additionally, [40] suggested that the hemibiotrophic *Mycosphaerella* banana pathogens require healthy banana plants for disease development, with [42] suggesting that the physiological state of detached leaves is not comparable with those on whole plants with an intact root system. Host gene expression in detached leaves during interaction with biotrophs has also been reported in *Arabidopsis* to closer reflect plant senescence [43]. For these reasons, bioassays were conducted using intact young leaves from 6 month old plants, with optimal temperature and humidity conditions employed during the experiment.

On the basis of BlastX search results against nr, Blast2GO annotation and assignment of unigenes to GO terms related to defense (Additional files 4 and 6) many unigenes potentially involved in plant Effector-Triggered Immunity (ETI) and PAMP-triggered Immunity (PTI) were identified across both genotypes. PTI is a branch of plant immunity which involves interactions between host pattern recognition receptor-like kinases (PRRs) and pathogen-associated molecular patterns (PAMPs) [44]. This branch involves a mitogen-associated protein (MAP) kinase cascade and WRKY transcription factors, and is responsible for resistance to the majority of potential pathogens. ETI [45], is based upon the co-evolution of plant resistance R protein receptors and specific pathogen effector molecules, responsible for disease resistance at the intra-specific level. Downstream signal transduction components can overlap between PTI and ETI, including an oxidative burst via the production of ROS, alterations in plant hormone production and MAPK signaling cascades.

### Expressed genes in Calcutta 4 and Cavendish Grande naine

*In silico* analysis of gene expression together with Blastx-derived annotation nomenclature revealed genes potentially involved in biotic stress responses in Calcutta 4. Numerous transcription factors were identified, which are typically involved in regulation of plant development, signaling and response to environment, amongst other roles. Many are also known to be involved in signal transduction and expression regulation of stress-responsive genes. Transcripts observed in infected leaf tissues in Calcutta 4 included a NAC domain protein and ethylene insensitive-like protein 4, a probable transcription factor acting as a positive regulator in the ethylene response pathway. Plant R-gene-mediated recognition of a biotrophic pathogen *Avr* gene product is associated with the hypersensitive response (HR), which results in programmed cell death at the site of infection to limit pathogen spread [46]. In inoculated Calcutta 4 plants, unigenes potentially involved in plant detoxification included a considerable presence of metallothionein-like proteins. These low molecular weight polypeptides sequester metal ions and are associated with regulation of intracellular redox potential and oxygen detoxification [47,48], protecting cells from damaging effects of reactive oxygen species (ROS). ROS generation may indicate HR activity, following pathogen infection and recognition. Four distinct types (MT 1 to MT4) are known in plants, according to distribution of cysteine residues. [49] reported isolation of types MT2 and MT3 in banana, with expression influenced in response to ethylene and metals. More recent examination has reported their abundance in *M. acuminata* Calcutta 4 [19]. Our study confirmed this, with MT2 and MT3 unigene contigs with considerable sequence depth. Superoxide dismutase enzymes (SODs) were similarly observed in infected Calcutta 4 leaves. Like metallothioneins, these also act as antioxidants, protecting plant cell components from oxidation by ROS. Glutathione-S-transferases were also represented. These may be involved in cell signaling pathways as well as in detoxification of products of oxidative stress during HR. Their expression in *M. fijiensis-M. acuminata* late stage compatible interactions has also recently been reported [23]. Reported peaks in accumulation of H_2_O_2_ and peroxidase activity in Calcutta 4 up to 10DAI with *M. fijiensis*[50] are consistent with our observations of abundance of transcripts for genes involved in ROS detoxification and HR during interaction with *M. musicola.* Phenylpropanoids in plants are involved in a number of defense responses, including biosynthesis of antimicrobial compounds such as phytoalexins and molecules involved in signaling. An abundance of gene transcripts for phenylalanine ammonia-lyase (PAL) was observed in Calcutta 4 following challenge with the pathogen. This enzyme catalyses a reaction in the phenylpropanoid biosynthetic pathway, with deamination of phenylalanine to cinnamic acid, leading to downstream synthesis of phytoalexins, as well as production of salicylic acid, a signal molecule involved in systemic acquired resistance (SAR). 4-coumarate-CoA ligase, another important enzyme in the biosynthesis of flavonoids and isoflavonoids, was also observed.

Blast nr-based functional annotation of expressed unigenes in Cavendish Grande Naine revealed an abundance with homology to transcripts poorly characterized according to the NCBI. However, where descriptions could be used as a guide, numerous unigenes in infected leaves were identified as potentially involved in plant responses to biotic stress. These included transcription factors, metallothionein-like proteins and superoxide dismutases (plant detoxification), 4-coumarate:coA ligase 2, cinnamic acid 4-hydroxylase and isoflavone reductase-like protein (phenylpropanoid pathway), and disease-related F-box protein and calmodulin binding protein (defense response). Examples of unigenes with fewer counts in infected leaves when compared with non infected tissues included WRKY transcription factor 17 and MAP kinase BIMK1 (defense signaling), putative callose synthase 1 catalytic subunit (plant callose synthesis), xyloglucan endotransglycosylase (xyloglucan-cellulose framework modification and strength of plant cell walls), endochitinases and putative chitinases (degradation of fungal cell walls), pathogenesis-related protein 1, F-box, wd40 domain protein, hypersensitive-induced response protein (plant defense), glutathione S-transferase 1 (plant detoxification), type III polyketide synthase 4, cinnamate-4-hydroxylase (phenylpropanoid pathway), CTR1-like protein kinase, ethylene receptor-like protein, ethylene response factor 11 and ethylene-responsive transcriptional coactivator (ethylene and defence signaling), respiratory burst oxidases (ROS signaling, signal transduction and cell death) and thaumatin-like proteins (degradation of fungal cell walls).

A number of distinct plant disease resistance *R*-gene families are recognized as involved in ETI and PTI, based upon protein domains and cell function. The most abundant class code for cytoplasmic receptor proteins with nucleotide binding site-leucine-rich repeat (NBS-LRR) domains [51]. In rice, approximately 400 NBS-LRR genes have been characterized, with 150 present in the *Arabidopsis* genome [52], and 89 identified in *M. acuminata* DH-Pahang [15]. Conservation of motifs within nucleotide-binding site leucine rich repeat domains has also enabled analyses of NBS-LRR R-gene family diversity across the genus (e.g. [38,53]). In the current study, 14 expressed NBS-LRR genes were identified through Blast analysis from both infected and non-infected leaf tissues for Calcutta 4 and 25 in Cavendish Grande Naine. Mapping of unigene contigs to gene models containing the NB-ARC domain in the reference *M. acuminata* genome identified 38 contigs mapping to 40 gene models in the case of Calcutta 4 transcriptome data and 43 Cavendish Grande Naine contigs mapping to 40 gene models (Additional file 1). Other known plant *R*-gene classes include extracellular LRRs anchored by transmembrane domains (receptor-like proteins), extracellular LRRs linked to cytoplasmic serine-threonine kinase domains (receptor-like kinases), intracellular serine-threonine kinases, and proteins with a coiled-coil domain anchored to the cell membrane. Blast analysis predicted numerous transcripts for these classes among the two genotype datasets.

Assignment to GO terms related to defense and Blast2go annotation (Additional file 3) provide a further level for mining candidate genes involved in host defense responses, complementing Blast nr-derived annotation. For example, a number of interesting unigenes in pathogen-inoculated Calcutta 4 leaf tissues were identified. These include glucan synthase components, which are associated with callose deposition in host cell walls during defense response, Rpm1 interacting protein 4, reported in plant defense involving R proteins RPM1 and RPS2, Mac perforin domain-containing proteins, which are associated with the salicylic acid (SA)-mediated pathway of programmed cell death, brassinosteroid insensitive 1-associated receptor kinase 1, which is involved in PTI and programmed cell death, and mlo-like protein 1, known to be involved in plant defense and cell wall strengthening.

### Quantitative real-time PCR analysis of gene expression

**Figure 4:**
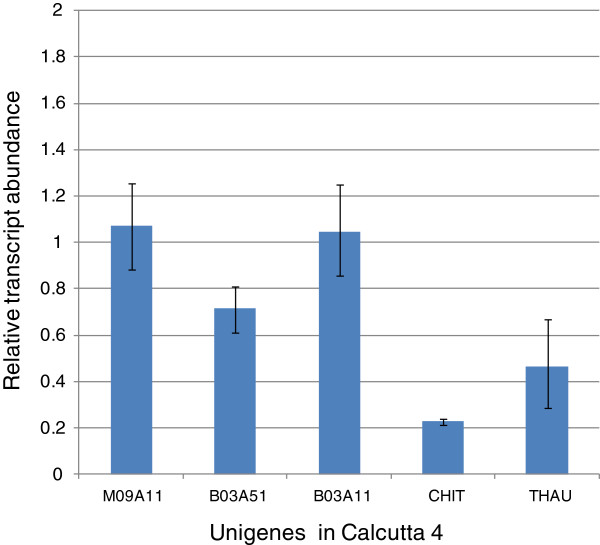
**Expression profiles in selected biotic stress responsive unigenes.** Quantitative real-time PCRs were performed to analyze expression in candidate unigenes in Calcutta 4 mRNA from pooled timepoints of 3, 6 and 9DAI, with triplicate samples of each. Expression analysis was conducted for LRR receptor-like serine/threonine-protein kinase RGA genes and SSR-containing defence-related unigenes. Normalization of expression was performed using an Elongation Factor gene for *M. acuminata* as reference. Bars represent the standard error of the mean of technical replicates for each biological sample.

Receptor-like protein kinases (RLKs) are transmembrane proteins with an N-terminal signal sequence, a specific receptor extracellular domain, and an intracellular C-terminal kinase domain. With over 600 RLKs characterized in *Arabidopsis*[54], only few are known to be involved in plant immunity [55,56]. Expression of LRR receptor-like serine/threonine-protein kinase RGA genes was evaluated in Calcutta 4, following alignment of 454 transcriptome data to annotated BAC sequences and specific primer design for mapped expressed genes. Quantitative real-time PCR revealed no significant change in expression in the three selected RGAs in relation to basal expression in non-inoculated controls (Figure 4). Although constant transcript levels during pathogen attack have been reported for different classes of host receptor R genes, where constitutive expression may mediate pathogen recognition and activation of signal transduction and defence responses, upregulation has been demonstrated in certain pathosystems in response to infection [57]. Expression analysis of potential host defence-related genes modulated during interaction with *M. musicola* was also conducted on randomly selected SSR-containing unigenes in Calcutta 4 (KOG descriptions: Pathogenesis related protein group 5, putative chitinase). Both genes were downregulated following pathogen challenge over the timecourse (Figure 4), with abundance of raw EST counts for each of the examined genes in inoculated and non-inoculated controls reflecting the changes observed with qRT-PCR data.

The overlaying of the 454 transcripts identified in the *M. acuminata* – *M. musicola* interaction onto gene models on the *M. acuminata* DH Pahang reference genome will facilitate ongoing validation of candidate gene expression via qRT-PCR. An in-depth understanding of the mechanisms by which R genes and downstream defence mechanisms function in *Musa* is necessary for development of novel strategies for durable resistance.

### Data mining against *M. fijiensis* gene models

Data mining for *M. musicola* pathogen transcripts amongst the pre-processed 454 sequence data derived from infected leaves was performed through genome mapping and alignment against the *Mycosphaerella fijiensis* v2.0 All Gene Models (transcripts) database. Based upon rDNA ITS sequence analysis, this species forms a monophyletic group with *M. musicola*[58]. Although pathogen transcript abundance is likely dependent on 454 depth of sequencing, a total of 10 unigenes from transcripts in pathogen-infected Calcutta 4 and 12 in Cavendish Grande Naine mapped to *M. fijiensis* gene models (Additional file 7). In addition to hypothetical proteins and no hits which were identified in both C4 and CAV, potential pathogen genes identified in transcriptome data for CAVI included a SAP family cell cycle dependent phosphatase-associated protein, two Hsp70 family proteins, a FAD binding domain protein, and a calcium channel. In the case of C4I data, positively mapped *M. fijiensis* gene models also included an extracellular protein 6 (Ecp6), and two 60S ribosomal proteins.

A total of seven Ecps have been identified in *Cladosporium fulvum* (syn. *Passalora fulva*)*,* which are secreted during interaction with tomato [59]. The *C. fulvum* effector protein Ecp6, crucial for virulence*,* contains carbohydrate-binding Lysin (LysM) domains that may be involved in binding to chitin released from fungal cell walls during the infection process [60]. Such PAMP-binding may prevent induction of plant basal defense responses. Orthologs of Ecp6 have been identified in EST and whole genome sequence data in a number of fungal genera. Within the genus *Mycosphaerella*, LysM-containing Ecp6-like proteins have been identified in *M. fijiensis*[59] and *Mycosphaerella graminicola*[59,61], indicating their likely presence in other members of the genus. Whilst homologues of the *C. fulvum* Ecp2 effectors have been described in *M. fijiensis* transcripts from *in vitro* culture [62], to date there have been no reports of *in vivo* Ecp effector homolog expression for the *Mycosphaerella* banana pathogens during host-pathogen interaction. Our identification of Ecp6 effector protein homolog transcripts in *M. musicola* may contribute towards identification of pathogen effectors and cognate disease resistance genes in *Musa*.

### Genic SSR markers

Microsatellite markers or simple sequence repeats (SSRs) are defined as tandem DNA repeats which are dispersed in both coding and non-coding regions along eukaryotic genomes. As molecular markers, they are typically somatically stable, polymorphic, co-dominant, and multi-allelic. Applications in *Musa* have focused on taxonomy (e.g. [63]), genotyping (e. g. [64]), and genetic map saturation (e.g. [65]). Gene-derived SSRs can be associated with functional genetic variation, as opposed to non-coding SSRs, with presence in transcribed regions potentially influencing gene function, transcription or translation [66]. Consequently they offer potential for marker assisted breeding, with markers either originating from a gene for a desirable phenotypic trait, or co-localizing with a particular quantitative trait locus. With such markers isolated from coding regions, conservation is also potentially greater, increasing transferability to related species (e.g. [67]). In comparison with other crops, relatively few SSR markers have been developed for the genus *Musa*, reflecting the limited sequence resources available until recently. Whilst the majority have been isolated from genomic libraries or BAC clones [35,63,68-71], only few gene-derived SSRs have been characterized (e.g. [72,73]).

**Figure 5:**
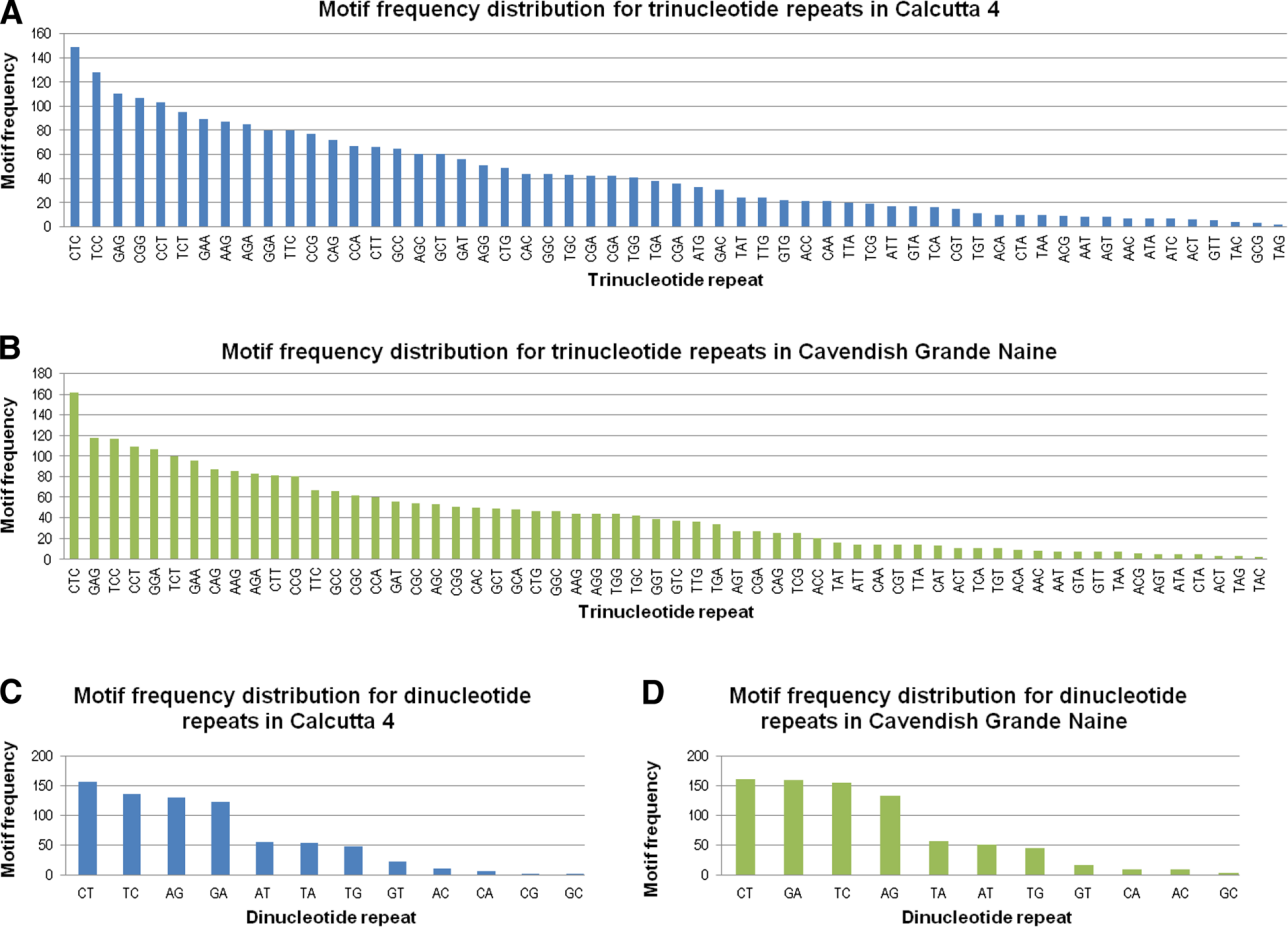
Motif frequency distribution for nucleotide repeats.

We screened the Calcutta 4 and Cavendish Grande Naine unigene datasets for the presence of SSR motifs. A total of 4,068 potentially PCR amplifiable genic-SSR loci were identified in the 36,384 Calcutta 4 consensus sequences, representing an EST-SSR frequency of 11.18%. Similar numbers were observed for the 35,269 Cavendish Grande Naine consensi, with a total of 4,095 SSRs, representing a frequency of 11.61%. In terms of SSR class distribution, for Calcutta 4 unigenes, trinucleotide repeats were the most abundant (61.4%), followed by di- (18.26%), tetra- (9.7%), hexa- (5.9%) and penta-nucleotide repeats (3.7%). Hepta-nucleotide repeats and above accounted for 1.04%. A similar distribution was observed in Cavendish Grande Naine unigenes, with trinucleotide core motifs representing 61.68% of repeats, followed by dinucleotides (19.4%), tetra- (8.6%), hexa- (5.8%) and pentanucleotides (3.5%). Again, heptanucleotide repeats and longer SSRs made up only 1.02% of the total. A predominance of trinucleotides in transcriptome sequence data is common [74,75], with the presence of such motifs in gene regions avoiding frameshift mutation introduction and changes at the protein level. As expected, the shorter the nucleotide core sequence, the greater the number of repeats present. For Calcutta 4 an average of 7.74 repeats were observed for di-nucleotide motifs, 4.99 for tri, 3.81 for tetra, 3.56 for penta, and 3.63 for hexa-motifs. Similarly for Cavendish Grande Naine there were an average of 8.55 repeats for di-, 4.99 for tri-, 3.75 for tetra-, 3.59 for penta-, and 3.70 for hexa- motifs. Motif frequency distribution for trinucleotide and dinucleotide core motifs, which were the most abundant repeats for both genotypes are shown in Figure 5.

The details of all 8,163 *M. acuminata* genic-SSR primers, including SSR motif, primer sequences, PCR amplification information, predicted gene function and *in silico* expression data are provided in Additional file 8. A subset of 95 potential defense-related gene-derived SSR loci, selected on the basis of Blast similarities and KOG descriptors, were validated for specific amplification and polymorphism across 20 diploid *M. acuminata* accessions, to complement previous work by our group [70]. A total of 73 (76.8%) amplified specific products of expected size from genomic DNA originating from the tested *M. acuminata* genotypes. Polymorphism was observed in 14 loci (14.7%), with alleles per polymorphic locus ranging from 3 to 8 and a total of 66 alleles scored across the polymorphic loci. Polymorphism information content ranged from 0.34 to 0.82, with an average of 0.63 (Table 2). This limited diversity can be expected for genic-SSR markers, as a result of DNA sequence conservation of transcribed regions [76,77].

**Table 2:** **Characteristics of polymorphic microsatellite loci isolated from *M. acuminata *Calcutta 4 and Cavendish Grande Naine unigene sets**

**Locus number**	**Unigene ID**	**SSR repeat motif**	**SSR locus length (bp)**	**Allelic Amplitude (bp)**	**Number of alleles**	**He**	**Ho**	**PIC Value**
locus821	musa_c4_small_c22955	CGG	14	170-180	3	0.61	0.52	0.53
locus945	musa_c4_small_c25361	TGACA	17	325-330	3	0.62	0.57	0.55
locus1284	musa_c4_small_c7084	AG	11	270-330	6	0.77	0.65	0.74
locus1412	musa_c4_small_c8798	TGC	15	175-190	3	0.39	0.14	0.34
locus1654	musa_c4_small_rep_c12642	CCT	16	170-200	5	0.70	0.71	0.66
locus2108	musa_c4_small_rep_c2099	GA	14	190-220	7	0.81	0.92	0.79
locus2061	musa_c4_small_rep_c1992	AG	19	190-200	5	0.70	0.52	0.66
locus2108	musa_c4_small_rep_c2099	GA	14	205-230	8	0.84	0.91	0.82
locus2112	musa_c4_small_rep_c21184	GTC	18	200-205	3	0.52	0.65	0.46
locus2306	musa_c4_small_rep_c2740	TG	13	170-185	4	0.74	0.38	0.69
locus2984	musa_c4_clusters_pos_c1484_1	TCCT	13	115-130	7	0.80	0.45	0.77
locus371	musa_cav_c18572	AT	12	350-360	3	0.56	0.54	0.48
locus2028	musa_cav_rep_c22610	CTT	17	280-290	4	0.70	0.75	0.65
locus2646	musa_cav_rep_c5186	TTC	18	200-220	5	0.73	0.30	0.68

Polymorphism was evaluated across 21 *M. acuminata* accessions. HE, expected heterozygosity under Hardy-Weinberg expectations; HO, observed heterozygosity; PIC, Polymorphism Information Content.

Our work represents a large scale development of new genic-SSR markers for application in genetic improvement, complementing the approximately 2,000 SSRs identified in BAC-end and sequence scaffolds of DH-Pahang [15]. With efforts underway towards development of segregant populations for traits of interest [78-80], these functional gene-based markers are applicable for association to trait loci and downstream marker-assisted selection, as well as evolution analysis, parentage assessment and general genotyping applications in breeding programs.

## Conclusions

This NGS-based investigation of the transcriptome in the *M. acuminata* – *M. musicola* interaction provides useful data on expressed genes during plant immune responses in this pathosystem, with 36,384 and 35,269 unigene sets identified, respectively, for contrasting *M. acuminata* genotypes Calcutta 4 and Cavendish Grande Naine. Genes were characterized according to Blast annotation, GO category assignment and Interpro-based domain identification. The data represent a global transcriptome level resource for *M. acuminata*, with identification of candidate genes expressed during infection contributing to our understanding of host defense mechanisms against this important pathogen. The recently published reference whole genome for DH-Pahang represents approximately 90% of the estimated genome size. Genome annotation updates will be facilitated by the availability and mapping of comprehensive sets of expressed gene sequences for this species. Our large scale characterization of genic-SSRs and marker development are a resource for application in genetic map enrichment, diversity characterization and downstream marker assisted breeding in *Musa*.

## Methods

### Plant material preparation

*M. acuminata* Calcutta 4 and Cavendish Grande Naine whole plants (*Musa* International Transit Centre accessions ITC0249 and ITC0654) were selected for transcriptome analysis based upon their known contrasting resistance to *M. musicola*. A total of 36 six month old plants were maintained in a greenhouse under a 12-h light/12-h dark photoperiod at 25°C and 85% relative humidity. Both infected and non-infected control plants were maintained under identical conditions. A strain of *M. musicola*, isolated from Cavendish Grande Naine leaf lesion sporodochia at Embrapa Cassava and Tropical Fruits, Brazil, was used for artificial inoculation of the abaxial surface of the youngest emerged leaf of each plant. Inoculation was conducted by spraying the entire leaf with a suspension of 2 × 10^4^ conidiospores ml^-1^, with addition of surfactant Tween 20 at 0.05%. Control samples comprised water-surfactant-treated leaves incubated under the same growth conditions. Sprayed leaves were covered with transparent plastic bags to ensure high humidity. Three independent replicates were collected for each sample.

**Figure 6:**
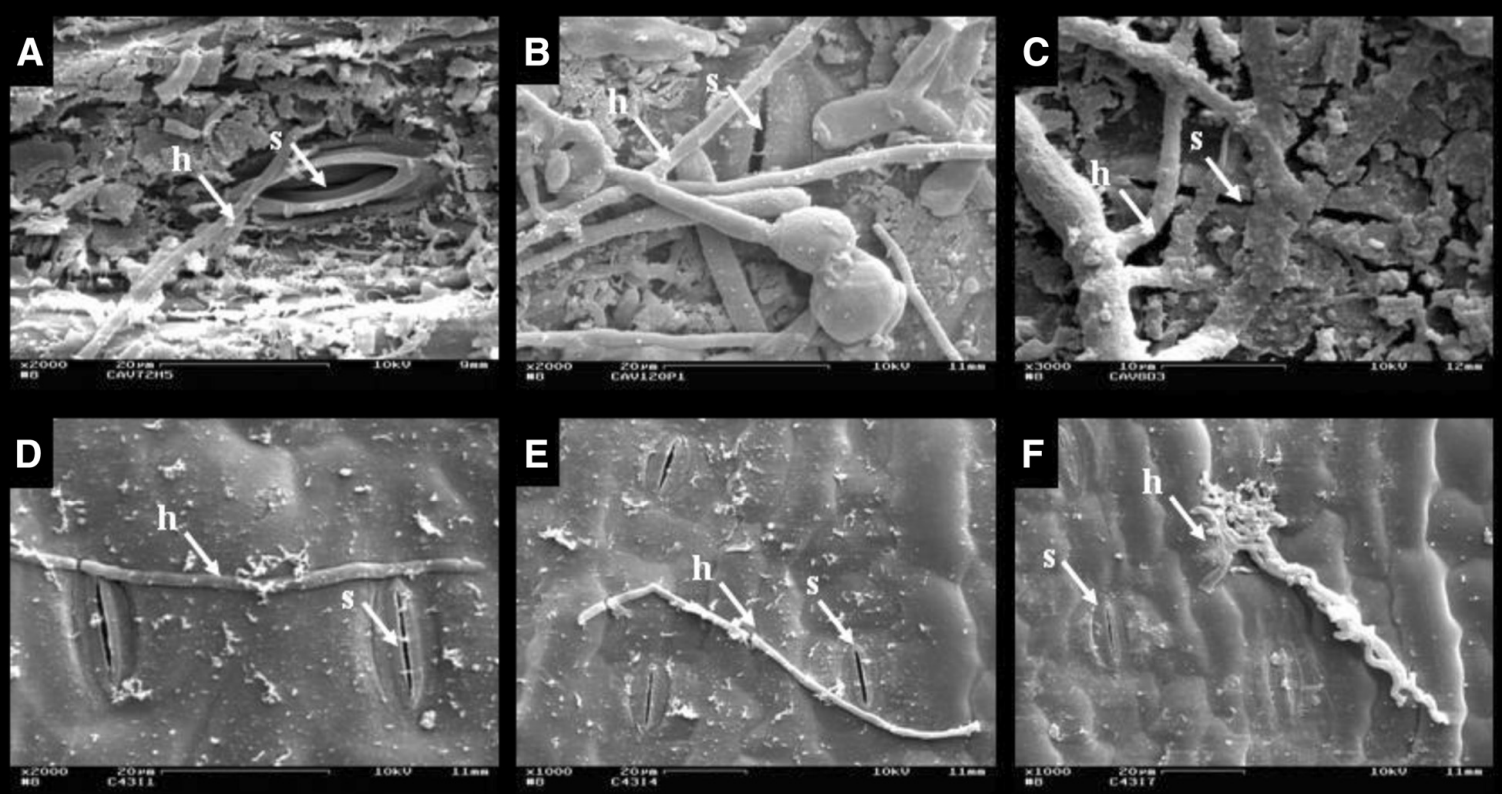
**Scanning electronic microscopy (SEM) observation of interaction of *Mycosphaerella musicola *with abaxial leaf surfaces of *Musa acuminata *genotypes.** Fungal germ tubes and hyphal growth were observed 3, 6 and 9DAI respectively, for Cavendish Grande Naine (**A**, **B**, **C**) and Calcutta 4 (**D**, **E**, **F**). Hyphal growth and penetration of stomatal openings was observed only in Cavendish Grande Naine 6DAI onwards. Abbreviations: DAI, days after inoculation; h, hypha; s, stoma.

Scanning electron microscopy was conducted to confirm *M. musicola* germination and infection, and used to determine time points for leaf harvesting. A total of three replicates per sample were prepared for analysis. Tissues were fixed for 2.5 h in a fresh solution of 0.05 M cacodylate buffer at pH 6.8, containing 2.5% glutaraldehyde fixer, washed in 0.1 M cacodylate buffer at pH 6.8, and postfixed for 1 h through addition of 2% osmium tetroxide to the buffer solution. Sample dehydration was conducted at 4°C for 20 minute periods with increasing concentrations of ethanol (10, 20, 30, 50, 70, 80, 90, 95 and 100%). Samples were then dried in a critical point drier (Emitech K850, Kent, UK), mounted on copper stubs and sputter coated with 20 nm gold particles. All samples were observed using a Zeiss DSM 962 Scanning Electron Microscope. Results showed that germ tubes and hyphae were visible at 3 days after inoculation (DAI), with hyphal growth over stomatal cells and penetration in Cavendish Grande Naine occurring at 6DAI onwards (Figure 6). Similar finding were reported by [49], who, investigating the *M. fijiensisM. acuminata* interaction, observed increasing stomatal penetration in Cavendish Grande Naine from 5DAI, up to a total of 11% of stomata penetrated 21DAI, and only a constant 0.95% of stomata infected for Calcutta 4, over the 21 day period. Based on these data, 1 g leaf samples from each genotype were collected at 3, 6 and 9DAI, immediately snap frozen in liquid nitrogen and stored at −80°C until further processing.

### RNA preparation

Total RNA from 1 g leaf samples was extracted using Concert® RNA Plant Reagent (Invitrogen, Carlsbad, CA, USA) and INVISORB Spin Plant RNA Mini Kit (Invitek, Hayward, CA, USA), according to manufacturer’s instructions. Total RNA was treated with DNase-free (Ambion, Austin, TX, USA) using 1.5 units/μg of total RNA. Quantification and integrity was assessed by using ethidium bromide-stained 1% agarose gels and Nanodrop ND-1000 spectrophotometry (Thermo Scientific, Waltham, MA, USA), with a a cut-off value of 1.8 for the A260 : 280 ratio. A total of 36 RNA samples were extracted from triplicate plant samples for infected leaves and non-inoculated control leaves at 3, 6 and 9 DAI from *M. acuminata* Calcutta 4 (C4I, C4NI) and *M. acuminata* Cavendish Grande Naine (CAVI, CAVNI). Four separate RNA pools were prepared for cDNA library construction, each comprising nine RNA samples (pathogen-challenged or unchallenged *M. acuminata* genotype, pooled timepoints 3, 6 and 9DAI, and triplicate samples).

### cDNA library construction and 454-sequencing

Messenger RNA isolation, full-length enriched cDNA library preparation and pyrosequencing was carried out by Eurofins MWG Operon (Ebersberg, Germany) from approximately 50 μg of each total RNA pool. Non-cloned libraries were prepared by random priming of polyA + mRNA and size fractioning. Emulsion PCR and sequencing were conducted according to Roche standard protocols using GS FLX technology and Titanium series chemistry. Each of the four libraries was sequenced in a ¼ segment of a picotiter plate.

### Sequence processing and annotation

#### Pre-processing

All sequence processing steps were performed iteratively using the software Est2assembly platform [81]. Raw 454 sequence files in SFF Format were converted to FASTA/QUAL-compatible sequences using the SFF Extract software (http://bioinf.comav.upv.es/sff_extract/). For sequence cleaning, adapter-ligated sequences were masked using SSAHA2 [82], and undesirable sequences (such as mitochondrial DNA, rDNA or contaminant-derived) identified for masking using BLAST2 [83]. Repetitive sequences were identified and masked using RepeatMasker version open-3.3.0 (http://repeatmasker.org).

#### Sequence assembly

Frequencies of DNA K-mers in sequence data was obtained using the software Nesoni version 0.89 (Victorian Bioinformatics Consortium), with sequence groups with distinctive K-mer frequencies clustered accordingly using WcdEST version 0.6.3 [84]. Sequence assembly of cluster group members into contigs and singletons was performed using the assembly program MIRA version 3.1 [85], with 454 technology-specific and expected k-mer coverage assembly parameters. Redundancy in assembled sequences originating from - K-mer groups was inspected using BLAST2 (option megablast).

In order to assess accuracy in assembly of the *de novo*-assembled unigene contig sequences for each genotype, alignment of reads plus their associated contig information was conducted against all annotated gene models in the *M. acuminata* ssp. *malaccensis* var. Pahang (DH Pahang) whole genome sequence (downloaded at http://banana-genome.cirad.fr) using the genomic mapping and alignment tool GMAP [86]. Alignment criteria comprised a minimum of 90% of the query sequence mapped on the genome and the percentage identity of the sequence relative to the mapped segment greater than 95%. Validation of unigene contigs that did not map to gene models on the reference *M. acuminata* genome was conducted through TBLASTX analysis against the NCBI EST (others) database (http://www.ncbi.nlm.nih.gov).

#### Sequence annotation

Functional annotation of known proteins/genes for *de novo*-assembled unique sequences from each K-mer group was conducted against the NCBI non-redundant protein (nr) database (http://www.ncbi.nlm.nih.gov) using the BLASTX algorithm, with a typical E-value cut-off of 10^-5^. Annotation of unigenes with no significant similarity to database sequences were further analyzed for protein domains with specific cell function using the software InterproScan version 4.8 [87]. Functional category assignment for each unique sequence was conducted using the Blast2GO program, classifying according to GO terms within molecular functions, biological processes and cellular components [88,89]. Comparative genomics analysis between Calcutta 4 and Cavendish Grande Naine unigene datasets was conducted through examination of common and distinct gene models in the *Musa* reference genome to which *de novo*-assembled unigenes were mapped.

For identification of potential fungal pathogen transcripts amongst the sequence data derived from infected leaves, pre-processed 454 reads were aligned against the closely related *Mycosphaerella fijiensis* v2.0 All Gene Models (transcripts) database using BWA [90] with default parameters. Counts according to reads mapped to *M. fijiensis* gene models were calculated using GenomicRanges (http://www.bioconductor.org).

### Simple sequence repeat (SSR) identification and marker development

Perfect SSRs in unigene sequences were identified through a computational search with the program Mreps version 2.5 (http://bioinfo.lifl.fr/mreps/). Detection required the presence of at least two repeating units (e.g. GC) spanning more than 10 bp. Primer pairs flanking each SSR locus were designed using the program Primer3 [91]. Genic-SSRs potentially related to biotic stress responses were selected based upon KOG classification and description. From a subset of 95 selected primer pairs, marker amplification and allele length polymorphisms were evaluated using 20 diploid (AA) *M. acuminata* accessions contrasting in resistance to Sigatoka diseases, as described previously [60]. A classical 10 bp molecular size marker was added to each gel to enable allele size estimation for PCR products run on denaturing 6% polyacrylamide gels using 7 M urea. Gels were silver stained according to standard protocols. Locus polymorphism was calculated using the Polymorphism information content (PIC) calculator (http://w3.georgikon.hu/pic/english/default.aspx).

### Quantitative real-time PCR analysis

Expression analysis of a number of selected candidate unigenes was conducted using quantitative real-time PCR (qRT-PCR). Two independent bioassays were performed, as described above, to enable comparison of gene expression in *M. acuminata* genotype cDNA pools for *M. musicola*-inoculated and non-inoculated controls, with three technical replicates for each biological replicate. RNA pools were prepared for cDNA library construction, each comprising nine RNA samples for the pathogen-challenged or unchallenged *M. acuminata* genotype (pooled timepoints of 3, 6 and 9DAI, with triplicate samples). Total RNA were digested with DNase (TURBO DNA-free™, Ambion USA) and converted into cDNA with Super-Script™ II RT and Oligo(dT)20 primer (Invitrogen, Carlsbad, CA, USA). Specific primers were designed using the program Primer3Plus [92], targeting Calcutta 4 defense-related unigene contigs containing SSRs and LRR receptor-like serine/threonine-protein kinase resistance gene analogs (RGAs) in Calcutta 4 and Cavendish Grande Naine. The RGA-targeting primers were designed by mapping all 454 pre-processed transcriptome reads onto annotated Calcutta 4 and Cavendish Grande Naine RGA-containing BAC clones [38], to enable primer design to exon and UTR regions. All RT-qPCR reactions were performed on an ABI 7300 Real-Time PCR System (Applied Biosystems, Foster City, CA, USA). Each PCR reaction mixture contained 1 μl of template cDNA, primer pairs (175 nM) and Platinum® SYBR® Green qPCR Super Mix-UDG w/ROX kit (Invitrogen, Carlsbad, CA, USA), according to manufacturer’s instructions. PCR amplification was conducted with 40 cycles of denaturation at 95°C for 15 s, primer annealing at 60°C for 30 s and extension at 60°C for 60 s. Gene expression normalization was performed against the internal reference gene coding for an elongation factor for *M. acuminata*. Amplification efficiencies, correlation coefficients, R2 values and relative gene expression (comparative C*t* method) were calculated using 7500 v.2.0.4 software (Applied Biosystem, Foster City, CA, USA). All primers used in the study are listed in Table 3.

**Table 3:** Primer sequences for qRT-PCR analysis of expression of SSR marker–derived genes associated with defense responses and BAC clone-derived LRR receptor-like serine/threonine-protein kinases

**Gene / contig abbreviation**	**KOG Gene description / BAC annotation**	**Primer Sequence Forward/Reverse**	**Amplicon Size (bp)**	**PCR efficiency (%)**	**Efficiency SD (+/−)**
THAU_musa_c4_small_rep_c3564	Pathogenesis related proteins, group 5, Thaumatin	CCGGTGGGACTAATTACAGG/ CAATTCGGATGTCAATGCAG	165	99	0,011
CHIT_musa_c4_small_c3092	Chitinase	CACCATCTCCTGCAAGCATA/ GCAGTCATTCCTCGTTGTCA	123	96	0,008
M09A11_454_Ma4140M09A11	Putative LRR receptor-like serine/threonine-protein kinase	CCAGCAACCACAACCCTAGT/ GATCTTTCAGGCCTCGTTTG	176	99	0,009
B03A51_454_Mac054B03A51	Putative LRR receptor-like serine/threonine-protein kinase	ATTCCATGGCTACGGACAAT/ TTCAGGCCTCGTTTGAACTC	192	107	0,005
B03A11_454_Mac054B03A11	Putative LRR receptor-like serine/threonine-protein kinase	CAGCAACCATAACCCCATTC/ AGGATCTTTCAGGCCTCGTT	168	105	0,011
EF	Elongation Factor	AACCCCCAAATATTCCAAGG/ AGATTGGCACGAAAGGAATC	107	102	0,013

Differential gene expression in *M. acuminata* Calcutta 4 inoculated with *M. musicola* was analyzed in relation to non-inoculated controls.

## Competing interests

The authors declare that they have no competing interests.

## Authors’ contributions

MANP participated in bioassays, RNA preparation for cDNA library construction and sequence data analysis. VOC participated in microsatellite marker characterization, validation and data analysis. FLE participated in bioassays, RNA preparation for cDNA library construction and sequence data analysis. CCT conducted bioassays and participated in RNA preparation for cDNA library construction. VCRA participated in microsatellite marker validation and data analysis. AB participated in QRT-PCR and data analysis. EPA participated in bioassays and editing of the manuscript. CFF participated in bioassays and editing of the manuscript. NFM participated in sequence data analysis and editing of the manuscript. RCT participated in sequence data analysis and editing of the manuscript. GJPJ participated in sequence data analysis and editing of the manuscript. OBSJ participated in sequence data analysis and editing of the manuscript. RNGM conceived the study, participated in bioassays, RNA preparation for cDNA library construction, sequence data analysis, and drafted the manuscript. All authors have contributed to, read and approved the final manuscript.

## Supplementary Material

Additional file 1 **Mapping data for *M. acuminata *Calcutta 4 and Cavendish Grande Naine unigene contigs aligned to *M. acuminata *DH Pahang all gene models.** Data shows all assembled contigs of *M. acuminata* Calcutta 4 and Cavendish Grande Naine generated through *de novo* assembly mapping to *M. acuminata* ssp. *malaccensis* var. Pahang (DH Pahang) whole genome sequence. Specific data is also presented for contigs mapped to cytoplasmic nucleotide binding site-leucine-rich repeat proteins, together with TBLASTX scoring hits obtained by comparison of all unmapped contigs against EST (others) database of GenBank at an E value < e-5. Click here for file

Additional file 2 **Example of *M. acuminata *Calcutta 4 unigene contigs mapping to single *M. acuminata *DH Pahang gene models.** Click here for file

Additional file 3 **Venn diagram showing the overlap between transcriptome unigene datasets (contigs and singletons) for the *M. acuminata * genotypes Calcutta 4 and Cavendish Grande Naine**. All gene models in the reference *M. acuminata* DH Pahang genome were used as a base for identification of common mapped genes. Click here for file

Additional file 4 **Consensus sequences and BLASTX analysis of assembled unigene contigs of *M. acuminata *Calcutta 4 and Cavendish Grande Naine.** Data represent the consensus sequences of assembled contigs of *M. acuminata* Calcutta 4 and Cavendish Grande Naine generated through *de novo* assembly, together with the three best BLASTX scoring hits obtained by comparison against nr database of GenBank at an E value < e-5. Click here for file

Additional file 5 **The most representative InterPro domains (IPR) observed across the assembled unigene contigs of *M. acuminata *Calcutta 4 and Cavendish Grande Naine.** Click here for file

Additional file 6 **Analysis of assembled unigene contigs of *M. acuminata * Calcutta 4 and Cavendish Grande Naine for Gene Ontology (GO) terms related to defense response.** Assembled contigs of *M. acuminata* Calcutta 4 and Cavendish Grande Naine were assigned to GO terms related to defense. Click here for file

Additional file 7 **Mapping data for *M. acuminata *Calcutta 4 and Cavendish Grande Naine pre-processed 454 sequence reads aligned to *Mycosphaerella fijiensis *v2.0 All Gene Models.** Click here for file

Additional file 8 **Sequence information of all the SSR primer pairs designed with Mreps and Primer3 for *M. acuminata *Calcutta 4 and Cavendish Grande Naine unigenes.** Data represent all the identified SSR loci, together with supporting information (SSR motif, primer sequences, PCR amplification information, predicted gene function and *in silico* expression data). Click here for file
