# Supplementary figures and images for: Analysis of the leaf transcriptome of Musa acuminata during interaction with Mycosphaerella musicola: gene assembly, annotation and marker development
Source: BMC Genomics. 2013 Feb 5;14:78. doi: 10.1186/1471-2164-14-78 (PMC3635893; doi:10.1186/1471-2164-14-78)

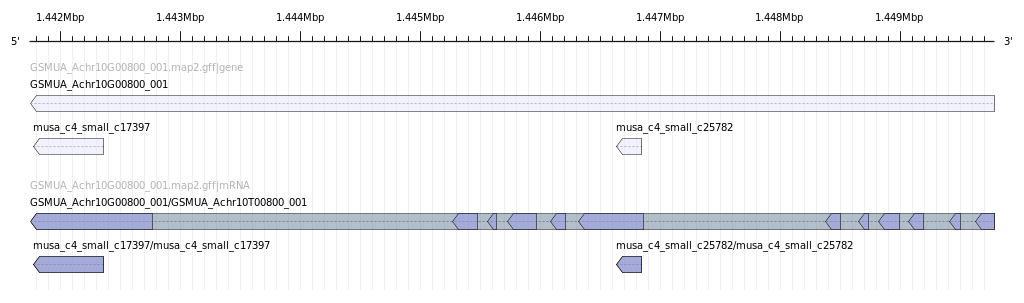

Supplement: Additional file 2 — Example of M. acuminata Calcutta 4 unigene contigs mapping to single M. acuminata DH Pahang gene models. [file 1471-2164-14-78-S2.png]

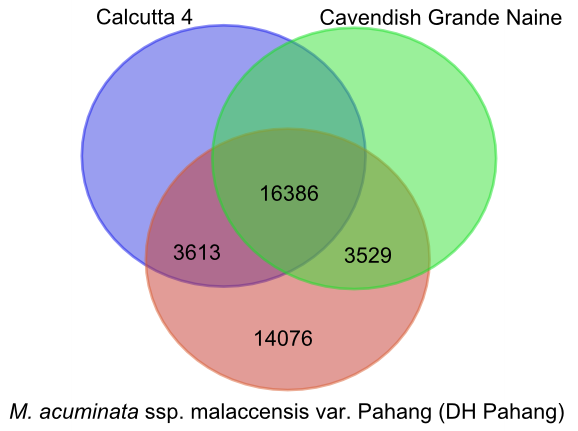

Supplement: Additional file 3 — Venn diagram showing the overlap between transcriptome unigene datasets (contigs and singletons) for the M. acuminata genotypes Calcutta 4 and Cavendish Grande Naine. All gene models in the reference M. acuminata DH Pahang genome were used as a base for identification of common mapped genes. [file 1471-2164-14-78-S3.docx]
